# The risk of colorectal cancer with symptoms at different ages and between the sexes: a case-control study

**DOI:** 10.1186/1741-7015-7-17

**Published:** 2009-04-17

**Authors:** William Hamilton, Robert Lancashire, Debbie Sharp, Tim J Peters, KK Cheng, Tom Marshall

**Affiliations:** 1 Academic Unit of Primary Health Care, Department of Community Based Medicine, University of Bristol, 25 Belgrave Road, Bristol, BS8 2AA, UK; 2 Department of Public Health and Epidemiology, University of Birmingham, Edgbaston, Birmingham, B15 2TT, UK

## Abstract

**Background:**

Colorectal cancer is generally diagnosed following a symptomatic presentation to primary care. Although the presenting features of the cancer are well described, the risks they convey are less well known. This study aimed to quantify the risk of cancer for different symptoms, across age groups and in both sexes.

**Methods:**

This was a case-control study using pre-existing records in a large electronic primary care database. Cases were patients aged 30 years or older with a diagnosis of colorectal cancer between January 2001 and July 2006, matched to seven controls by age, sex and practice. All features of colorectal cancer recorded in the 2 years before diagnosis were identified. Features independently associated with cancer were identified using multivariable conditional logistic regression, and their risk of cancer quantified.

**Results:**

We identified 5477 cases, with 38,314 age, sex and practice-matched controls. Six symptoms and two abnormal investigations (anaemia and microcytosis) were independently associated with colorectal cancer. The positive predictive values of symptoms were: rectal bleeding, positive predictive value for a male aged ≥ 80 years 4.5% (95% confidence interval 3.5, 5.9); change in bowel habit 3.9% (2.8, 5.5); weight loss 0.8% (0.5, 1.3); abdominal pain 1.2% (1.0, 1.4); diarrhoea 1.2% (1.0, 1.5) and constipation 0.7% (0.6, 0.8). Positive predictive values were lower in females and younger patients. Only 27% of patients had reported either of the two higher risk symptoms.

**Conclusion:**

Most symptomatic colorectal cancers present with only a low-risk symptom. There is a need to find a method of identifying those at highest risk of cancer from the large number presenting with such symptoms.

## Background

Over 35,000 colorectal cancers were diagnosed in England in 2004 [1]. Survival in the UK is worse than in other European countries and the US, partly because of later presentation in the UK [2]. Delays in presentation to medical care and diagnosis have been observed in several studies [3].

Early diagnosis may reduce mortality. The relative risk of death from colorectal cancer is between 0.67 and 0.85 following faecal occult blood screening [4]. A large UK prospective study of screening in half a million patients aged 50 to 69 years found 2% to be faecal occult blood (FOB) positive, of whom 11% had colorectal cancer [5]. In 2006, a UK national screening programme was introduced using FOBs in people aged 60 to 69 years [6]. However, only around a quarter of colorectal cancers are likely to be detected in such a screening programme, because most become symptomatic after this age, the sensitivity of the test is only 60%, and almost half of eligible patients decline screening [5,7]. Thus, most colorectal cancers will continue to present with symptoms [8,9].

General practitioners (GPs) gain relatively little experience in the diagnosis of colorectal cancer because the incidence is about one per GP per year, and only a proportion of these are diagnosed in primary care [10]. In addition, the relevant symptoms occur frequently in association with benign conditions, which are also much more common. At least half of colorectal cancers only manifest low-risk symptoms such as constipation or abdominal pain, and there is no intermediate test to identify those particularly likely to harbour cancer (unlike, for example, a chest X-ray in possible lung cancer). Moreover, the main diagnostic test for suspected colorectal cancer is colonoscopy, with a small but important complication rate.

The UK Referral Guidelines for Suspected Cancer [11], which were updated in 2005, have a weak evidence base, concentrate on typical presentations of cancer and may even delay diagnosis in patients with atypical presentations [12]. Since publication of these guidelines, several studies have reported risk estimates for the common symptoms of colorectal cancer when presented to primary care. Rectal bleeding studies reported positive predictive values (PPVs) ranging from 2% to 6% [13-16]. Other studies have examined change in bowel habit [14], anaemia [14,17], abdominal pain and loss of weight [15].

However, few studies have examined the effect of age or sex. One calculated a steep increase in PPVs for rectal bleeding and change in bowel habit with age, in parallel with the increasing incidence of colorectal cancer [14]. Others showed a smaller rise [15] or almost none [18]. Males had higher PPVs for most clinical features, reflecting the higher incidence in men. Differences with age and sex are important; current guidance for referral ignores age (other than setting a minimum age of 60 years for referral with rectal bleeding or motility symptoms, and 40 years when both are present) and sex (apart from a lower threshold haemoglobin value for women). Most other countries have no specific guidance for identification of symptomatic colorectal cancer, although screening is recommended generally from the age of 50. We therefore studied the symptoms of colorectal cancer in a large electronic dataset to calculate risk estimates in narrow age bands and in both sexes.

## Methods

Data were provided by The Health Improvement Network (THIN), a database of electronic medical records uploaded from general practices using the VISION computer system. It is an offshoot of the General Practice Research Database (GPRD), restricted to practices within the GPRD using that specific computer technology. The records contain patient characteristics, all prescriptions, consultations, diagnoses and primary care investigations. There are currently 2.2 million active patients in over 300 practices: 4.7 million patients when historical data are included. The THIN database is subject to frequent internal quality checks, with any practices failing to maintain adequate quality standards removed from the database.

### Identification of cases and controls

Cases were patients aged 30 years or older with a diagnosis of colorectal cancer between January 2001 and July 2006 and at least 2 years of full electronic records before diagnosis. Up to seven controls per case were selected (the maximum allowed within THIN regulations), using a computerised random number sequence. Controls were matched for practice, sex and age, but had not had colorectal cancer: they also had at least 2 years of records before the index date (the date of diagnosis of their matched case). The full date of birth was unavailable to protect anonymisation; however, the year of birth was available. Where possible, controls were matched to the same year of birth in years as cases; if no controls in this year were available, they were matched to within 1 year, then 2 years and so on up to a maximum of 5 years. THIN staff identified and provided complete records for all cases and controls.

### Definition of variables

From a review of the literature, 23 candidate variables (features) were identified, either a symptom, or an abnormal primary care investigation, or a predisposing risk marker such as obesity. We also identified codes for irritable bowel syndrome as a potential misdiagnosis. For some symptoms we were also able to take advantage of the availability of data on related prescriptions, for example, prescriptions for antidiarrhoeals and laxatives were obtained as possible surrogates for the relevant symptoms, and similarly antispasmodic drugs for irritable bowel syndrome. Features were designated as new if there were no similar symptoms or prescriptions observed previously in the 2 years before the index date. Haemoglobin values were categorised into 1 g/dl bands, and microcytosis defined as a mean red cell volume ≤ 80 fl. Weight loss was calculated from the change between the last recorded weight and the highest weight in the previous 2 years, separated into two categories: ≥ 10% weight loss or 5% to 10% weight loss. Patients were assigned to their maximum weight loss category. Obesity was defined as a body mass index > 30 kg/m^2 ^within 2 years of the index date. Diabetes was considered to be present if it had ever been diagnosed.

### Analysis: identification of variables independently associated with cancer

The main method of analysis was conditional logistic regression. For the variables that also had prescriptions as surrogates, preliminary analyses compared the odds ratio for a prescription without a record of the feature with the odds ratio for the feature itself. As these were similar for all three of this type of variable, constipation, diarrhoea and irritable bowel syndrome, the relevant pairs of variables were merged.

All variables with a univariable association with cancer significant with a *P *value < 0.1 were entered into a staged multivariable analysis. The first stage of the multivariable analysis grouped clinically related variables together. These were: intestinal motility features (constipation, diarrhoea, change in bowel habit and flatulence); pain features (irritable bowel syndrome, or prescription of an antispasmodic and abdominal pain); bleeding features (rectal bleeding, anaemia and microcytosis); systemic features (weight loss and thrombo-embolism); obesity features (diabetes and obesity). Only variables with a *P *value < 0.05 within their group were considered for the final model.

### Analysis: estimation of PPVs

Age was stratified into four bands: 30 to 59 years (there were too few cancers in this band for meaningful sub-division) and 10-year bands thereafter, up to 80+ years. For each age/sex/feature combination we created a 2 × 2 table and calculated the positive likelihood ratio and its 95% confidence intervals. We used Bayes' theorem (posterior odds = prior odds × likelihood ratio) to estimate PPVs [19]. The prior odds were derived from national incidence rates stratified by age and sex for 2003 (the middle year of our cohort), in that an annual incidence of, for example, 5 per 1000, is the equivalent of 199 to 1 odds against having cancer diagnosed in the next year [20]. For males, the annual incidence rates used were: aged 30 to 59 years, 0.026%; 60 to 69 years, 0.19%; 70 to 79 years, 0.35% and 80+ years, 0.43%. Female rates for the same age groups were: 0.02%, 0.11%, 0.21% and 0.27%.

With the 5000 cases available, there was > 99% power to detect 5% having a particular feature, compared with 1% amongst controls. Such a difference was viewed in advance as being potentially clinically useful, and from a previous primary care study was considered possible [15]. Ethical approval was obtained from the London Multicentre Research Ethics Committee.

## Results

**Figure 1:**
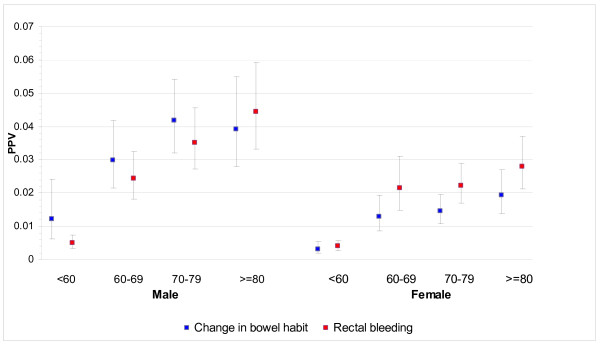
**Positive predictive values (PPVs) for rectal bleeding and change in bowel habit by age and sex**.

**Figure 2:**
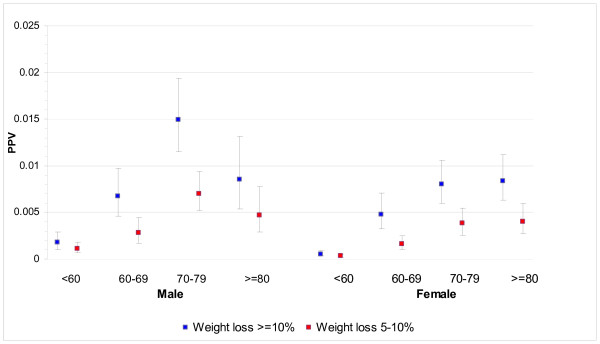
**PPVs for weight loss by age and sex**.

**Figure 3:**
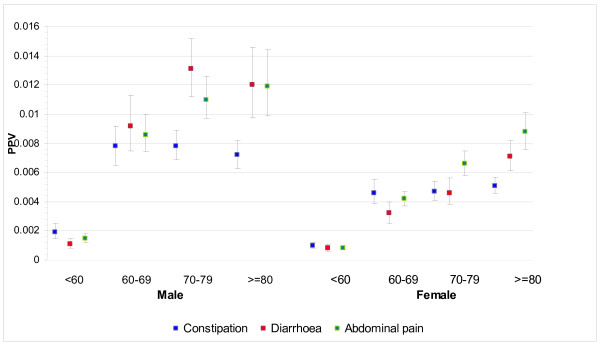
**PPVs for constipation, diarrhoea and abdominal pain by age and sex**.

THIN staff identified 5477 cases from 317 practices and were able to match 38,314 controls, with only seven very elderly cases having fewer than seven controls available. 36,925 (96.4%) controls were matched to the same year of birth, and 1150 (3%) to the adjoining year, leaving only 239 controls 2 to 5 years different in age. 2911 (53.1%) of the cases were male. The median (inter-quartile range) age at diagnosis of cases was 72 years (63, 79), with males slightly younger: male median 71 years (62, 78) and female 73 (63, 81): *P *< 0.001, Wilcoxon test. The frequencies of each clinical feature are shown in Table 1. Less than 2.5% of cases or controls had an abnormal rectal examination (15 cases, 2 controls), abdominal masses (86 cases, 19 controls), a positive FOB (7 cases, 2 controls), or thrombo-embolism (24 cases, 74 controls). These (rare) variables were omitted from further analyses. The final multivariable model is shown in Table 2. PPVs for the six symptoms independently associated with cancer, stratified by age and sex, are shown in Figures 1, 2 and 3, with scales varying for the high, medium and low-risk symptoms, respectively.

**Table 1:** Frequency of clinical features in cases and controls.

	**Cases** **(*N *= 5477)**		**Controls** **(*N *= 38,314)**		**Positive likelihood ratio (95% CI)**
	
**Clinical feature**	**Number with this feature**	**Percentage with this feature**	**Number with this feature**	**Percentage with this feature**	
**Symptoms**					

Constipation	1477	27.0	4051	10.6	2.6 (2.4 to 2.7)

Diarrhoea	988	18.0	2171	5.7	3.2 (3.0 to 3.4)

Change in bowel habit	615	11.2	375	1.0	5.5 (5.2 to 5.8)

Rectal bleeding	853	15.6	460	1.2	6.0 (5.7 to 6.3)

Weight loss					

5.0–9.9%	210	3.8	852	2.2	1.6 (1.4 to 1.8)

≥ 10%	351	6.4	678	1.8	2.9 (2.6 to 3.1)

Abdominal pain	1629	29.7	3121	8.1	3.5 (3.3 to 3.7)

**Investigations**					

Haemoglobin < 12.0 g/dl	1424	26.0	1803	4.7	4.4 (4.2 to 4.6)

Mean red cell volume < 80 fl	363	6.6	923	2.4	2.8 (2.4 to 3.1)

**Diagnoses and risk factors**					

Irritable bowel syndrome	135	2.5	325	0.8	2.4 (2.1 to 2.8)

Diabetes	626	11.4	3679	9.6	1.2 (1.1 to 1.3)

Obesity	510	9.3	3510	9.2	1.0 (0.93 to 1.1)

Note: the positive likelihood ratio is the likelihood of having a feature when one is a case, divided by the likelihood of having the feature when one is a control.

**Table 2:** Independent associations between clinical features and colorectal cancer in the final multivariable conditional logistic regression model.

**Clinical feature**		**Odds ratio (confidence interval)**
**Symptoms**		

Rectal bleeding		20 (17 to 23)

Change in bowel habit		14 (12 to 17)

Abdominal pain		3.9 (3.6 to 4.3)

Diarrhoea		2.4 (2.1 to 2.7)

Constipation		2.1 (1.9 to 2.3)

Weight loss		

	5.0–9.9%	1.2 (0.99 to 1.5)

	≥ 10%	2.5 (2.1 to 3.0)

**Investigations**		

Haemoglobin (g/dl)		

	12.0–12.9	1.7 (1.5 to 1.9)
	
	11.0–11.9	2.8 (2.4 to 3.2)
	
	10.0–10.9	5.9 (4.8 to 7.2)
	
	9.0–9.9	9.3 (7.1 to 12)
	
	< 9.0	18 (14 to 25)

Mean red cell volume < 80 fl		6.5 (5.3 to 7.9)

Note: associations adjusted for each other; all features have *P *< 0.001.

## Discussion

This large study shows eight clinical features presenting in primary care to be independently associated with the subsequent diagnosis of cancer. All were previously known, but the precise risk they confer had not been quantified with such precision, nor have they been clearly differentiated across ages and sexes. In general, the PPVs rose with age, particularly after the age of 60, and were higher in males. However, the absolute risk was relatively low (below 2%) for all ages and both sexes for constipation, diarrhoea, abdominal pain and loss of weight. Only change in bowel habit (a term that GPs use when the pattern of constipation or diarrhoea is particularly suggestive of colon cancer, or is accompanied by additional symptoms) and rectal bleeding had PPVs of 2% to 5% (and these higher PPVs were largely seen in males).

### Strengths and weaknesses

This is the largest primary care study to examine all colorectal cancer symptoms. Its size has allowed estimates of PPVs for relatively small age bands in either sex, and with reasonably narrow confidence intervals. Furthermore, the THIN database is representative of the UK as a whole, so our results should be generalisable. However, there are weaknesses too. We could not confirm cancer diagnoses, although it is unlikely that such an important diagnosis would have been entered erroneously particularly often. We also could not examine histology or staging, as these are only rarely recorded on primary care systems. The study relied upon good recording of symptoms. This problem is not quite as important as it appears, as under-recording of symptoms occurring proportionately in both cases and controls would not alter the likelihood ratios. Only if under-recording was especially prevalent in one group would a bias arise. It is impossible to know whether cases or controls would be more prone to under-recording. However, the PPVs are very similar to those derived from paper records [15] (these may of course also suffer from differential recoding of symptoms) and to the two previous studies using electronic records [13,14]. One further limitation was the study design: by matching for age and sex, we could not examine these directly. Even so, the dataset was large enough to allow age-sex stratified analyses and estimation of PPVs.

### Comparison with previous literature

Only three papers have used primary care data to estimate risks of colorectal cancer across the ages and between the sexes, although none with the size or precision of this one. The first examined GP records directly and showed an approximate tripling of PPV for rectal bleeding for those aged over 70 compared with those aged 40 to 69 years [15]. Two studies using electronic primary care records reported a rise in the PPV for rectal bleeding across 10-year age bands. This was also seen in the study reported here.

There is much less previous literature for other symptoms: one study gives similar figures for change in bowel habit [14], and the other again showed an approximate tripling of PPV for constipation, diarrhoea and abdominal pain between patients aged 40 to 69 years and those aged over 70 [15].

### Relevance of the findings

It is important to use primary care data to provide evidence for primary care decisions such as when to refer for investigation. Current referral guidelines essentially ignore age (other than giving a minimum age for referral) and sex (except giving a different threshold of haemoglobin for referral). As the incidence of cancer rises steeply with age, being 16 times higher in the oldest male age group and 13 times higher in the oldest female group when compared with the youngest groups, PPVs could have been expected to differ by as much. They do not, as the likelihood ratios fell with age. This presumably reflects the increasing prevalence of benign causes of colorectal cancer symptoms with age.

Two groups of symptoms emerge from the results. The highest PPVs are for rectal bleeding and change in bowel habit. For men over 60 years, rectal bleeding PPVs ranged from 2.4% to 4.5%; most would agree these figures are high enough to warrant investigation. For women, the figures were lower, but still in the 2% to 3% range. Change in bowel habit is less simple: GPs contributing to THIN must have been using this term very differently from the separate terms of constipation and diarrhoea, in that the PPVs for change in bowel habit were considerably higher. We cannot know what features led GPs to write change in bowel habit in the notes in preference to the specific motility symptoms, however, from our results it is clear that they were identifying a riskier feature, and one that warrants investigation.

In contrast, the PPVs for constipation, diarrhoea, abdominal pain and loss of weight were all below 1.5%, confirming that they are low-risk symptoms, at any age and in either sex. Indeed, this is the first study to show that the risk of colorectal cancer increases with increasing weight loss. This is no surprise (and is a testament to the quality of THIN data). However, these four symptoms are not 'no-risk' symptoms. This creates a problem for design of referral strategies. The high-risk symptoms of rectal bleeding and change in bowel habit were only recorded in 15.6% and 11.2% of cases, respectively. Even allowing for possible under-recording, it is highly likely that only a minority of patients with colorectal cancer have a high-risk symptom. The majority, with only a low-risk symptom, could have their diagnosis expedited in a number of different ways. Firstly, scoring systems such as the CAPER score may refine the risk in such patients [21]. Secondly, it is possible that biomarkers may be identified with adequate performance characteristics for use in such a population. Thirdly, early work suggests that measurement of rectal DNA may allow patients at high risk to be selected for investigation [22]. Without one of these initiatives, or a combination of them, patients with a low-risk symptom will continue to be at risk of delayed diagnosis, and possible emergency presentation [23].

## Conclusion

Our results generally support the National Institute for Clinical Excellence referral guidance with respect to symptoms. This is in contrast to the guidance for anaemia, with an unacceptably high PPV at the current threshold recommended for referral [17]. The choice of 60 as a starting age is supported by our findings. Colorectal cancer can occur in younger age groups, but the risk with particular symptoms is very low [24]. Our findings strengthen the view that rectal bleeding carries a high enough risk to warrant investigation irrespective of other symptoms. Current guidance requires persistence of bleeding for 6 weeks, or accompanying diarrhoea [11]. In our opinion, these additional requirements are unnecessary given no study has reported a risk below 2% for rectal bleeding *per se*. Even so, there remains a major gap within current guidance: the patient with the low-risk, but not no-risk, symptom. This is important, and suggests a direction for research efforts aimed at reducing mortality from colorectal cancer.

## Abbreviations

FOB: faecal occult blood; GP: general practitioner; GPRD: General Practice Research Database; PPV: positive predictive value; THIN: The Health Improvement Network

## Competing interests

The authors declare that they have no competing interests.

## Authors' contributions

RL constructed the database and performed the analyses guided by WH and TM. All six authors contributed to the design and supervision of the study, and all have read and approved the manuscript. The first draft and revisions were written by WH, incorporating suggestions from the other authors throughout.

## Pre-publication history

The pre-publication history for this paper can be accessed here:


